# Mental Distress and Psychological Disorders Related to COVID-19 Mandatory Lockdown

**DOI:** 10.3389/fpubh.2021.585235

**Published:** 2021-03-26

**Authors:** Ameer Kakaje, Ammar Fadel, Leen Makki, Ayham Ghareeb, Ragheed Al Zohbi

**Affiliations:** ^1^Faculty of Medicine, Damascus University, Damascus, Syria; ^2^Faculty of Medicine, Aleppo University, Aleppo, Syria; ^3^Department of Experimental Surgery, McGill University, Montreal, QC, Canada

**Keywords:** COVID-19, lockdown, psychological distress, posttraumatic stress disorder, mental disorders, conflict, developing country, Syria

## Abstract

**Background:** Lockdown restrictions due to COVID-19 have affected many people's lifestyles and ability to earn a living. They add further distress to the lives of people in Syria, who have already endured 9 years of war. This study evaluates distress and the major causes of concerns related to COVID-19 during the full lockdown.

**Methods:** Online questionnaires were distributed using SPTSS, K10, and MSPSS which were used with other demographic, war- and COVID-19-related questions that were taken from The (CRISIS) V0.1 Adult Self-Report Baseline Form.

**Results:** Our sample included 5,588 with the mean age of 26.84 ± 7.815 years. Of those, only one case of COVID-19 was confirmed. Over 42.7% had two or more positive PTSD symptoms, 42.6% had moderate or severe mental disorder, but only 14.9% had low social support. Higher PTSD and K10 scores overall were seen in female participants and with most of war variables (*P* < 0.05). Relationships with the partner being negatively affected and distress from a decline in ability to work and provide food were the most prominent.

**Conclusions:** The indirect effects of COVID-19 are far more than that of the pathogen itself. A reduced ability to earn and to provide food were the main concerns indicated in this study. Relationships deteriorated in participants with high K10 and PTSD scores who also had more symptoms and used more hypnotics in the last four weeks. Smoking patterns were not related to K10 and PTSD. Social support played a role in reducing stress, but when relationships were affected, lower support was observed.

## Introduction

Further to the ramifications of war which the Syrian population has been experiencing since 2011, the spread of the COVID-19 virus has created additional challenges. With the new restrictions imposed by the government in Syria, the country was in full lockdown despite having few confirmed cases; all non-essential business, schools, universities, parks, mosques, churches, and other areas of common gathering were closed. A forced lockdown was announced from 6 p.m. to 6 a.m. on weekdays and 12 p.m. to 6 a.m. on weekends. This potentially made it even harder for Syrians to cope with the underlying stress caused by years of insecurity, fear, and loss. While institutions and governments around the world designated specific hotlines, projects and support platforms for citizens coping with the stress of changes to their life brought by the pandemic (1–4), such measures in Syria did not take place. The reason for this could be attributed to the stigma regarding mental health, which is highly prevalent in most developing countries (5) but also to the difficult nine years of war the country had been experiencing, making any mental health programs now appear unreasonably out of context.

Previous literature on outbreaks has focused on the physical health consequences of the disease and less on the mental health sequela that social distancing can generate. However, disasters, whether they are natural, man-made or industrial, have an impact on the social structure of the community and therefore strongly impact the mental health of these communities (5). This impact can cause and aggravate medical conditions such as post-traumatic stress disorder (PTSD), depression, substance abuse, and a broad range of maladaptive and harmful behaviors such as child and domestic abuse (6). For instance, after the hurricane of Maria in Puerto Rico, the country had 25 suicides every month during the three months following the hurricane and 19 suicides per month in the eight following months, which were an increase from the baseline before the hurricane (7). As for PTSD, it was diagnosed in 30–40% of people surviving a disaster compared to the 8% prevalence in the general population (7).

Studies have tried to understand the impact of disease outbreaks on the mental health of those affected, proving that survivors of diseases, like SARS, can suffer from elevated stress and worry, even one year after the disease outbreak (8). While it is very important to understand the ramifications of disease outbreaks on the mental health of the community affected directly by the disease, the impact of the crisis goes beyond those who acquire the disease and affect even the healthiest members of the community (9). Some risk factors that make individuals more prone to have impaired wellbeing and quality of life during COVID-19 have been identified such as fears of infection, being lonely, boredom, frustration, and pervasive anxiety while others have been identified as protective factors including resilience and social support (10). These risk factors may also be related to negative outcomes such as suicide. It is suggested that these aspects might be from neglect and abuse since childhood (11).

Our study aims to understand that impact in the context and background of war and evaluates how disease outbreaks and war have significant consequences on mental health. In this study, we assess mental health during COVID-19 by asking participants direct questions related to the outbreak and its effect on life, and indirectly by measuring post-traumatic stress disorder (PTSD), social support, and mental distress. This study used the same methods adopted by a previous study, conducted one year before to allow comparisons (12). We hypothesize that PTSD symptoms and mental distress from living in war-torn Syria have increased during COVID-19 and that social support minimized it. We also speculated that COVID-19 related variables are similar to the effects of war on mental health.

## Methods

### Sampling

We conducted a cross-sectional study across Syria from April 6 to April 13, 2020. Online surveys were distributed in Arabic to participants from several Syrian governorates. The study only included participants who were living within Syria. Participants who answered key questions and lived in Syria during COVID-19 lockdown and generally in Syria in the last 9 years were included. The questionnaires were posted online twice each day at 10 a.m. and 10 p.m. in online Social Media groups that were concerned with different topics to cover the widest possible population.

### Questionnaires

#### Socioeconomic Status (SES)

Socioeconomic status (SES) was assessed by asking whether the place where the participants lived was owned, rented, or whether they were living with friends or accommodation provided by the government. We also asked whether the family income was adequate for essentials, or allowed them to buy more items.

#### Screening for Mental Disorder

An Arabic version of Kessler 10 + LM (K10 + LM) was used to screen and measure the severity of psychological distress (13–15). K10 is a self-reported measure that enables the assessment of anxiety and depression in the last 4 weeks, with scores ranging from 10 to 50, and each question has five possible responses. The answers score from one when replying “none of the time” to five when replying “all of the time.” Higher scores are indicative of higher levels of psychological distress. The severity of mental distress is divided into four levels: scores 10–19 will be likely well, scores 20–24 will likely have a mild disorder, scores 25-29 will likely have a moderate disorder, and finally scores 30 and above will likely have a severe disorder.

#### Social Support

We used the Arabic version of the Multidimensional Scale of Perceived Social Support (MSPSS) (16, 17) to assess the social support from friends, significant others, and family with four questions for each source. We used total mean scores (not means of each individual question) for comparisons with other variables. The norms will likely vary according to culture, nationality, age, and gender. The mean scores for the total or individual subscale are divided into 3 categories: 1 to 2.9 to be considered low support, 3 to 5 to be considered moderate support, and 5.1 to 7 to be considered high support.

### PTSD

The Screen for Posttraumatic Stress Symptoms (SPTSS) tool of diagnostic and statistical manual of mental disorders (DSM) IV was used. It contains three clusters of avoidance, arousal, and re-experience. The first two responses of “Not at all” and “1 or 2 times” represent the score 0 and the other responses represent the score 1. When scoring three or more on avoidance, two or more on arousal, and one or more on re-experience, that cluster is considered positive. Although it is based on DSM IV, it is somewhat close to what is used in the International Classification of Disease 11 (ICD-11) criteria and is reliable when screening for PTSD. SPTSS is a brief screening method that is not based on a single trauma model which helps to identify individuals who have high levels of SPSS symptoms and are not linked to a specific event.

### COVID-19 Questions

We used questions that involved COVID-19 from The Coronavirus Health Impact Survey (CRISIS) V0.1 Adult Self-Report Baseline Form (18), and we translated them and added extra questions. CRISIS is a self-reported baseline current form, and we only used some of the questions. Questions involved Health/Exposure status in the last 2 weeks, distress from COVID-19, feeling new symptoms that could not be attributed to allergies, distress from different aspects, smoking, relationships, and earning money being affected. These questions with their answers are demonstrated in Table 1.

**Table 1:** Characteristics of responders and their responses to COVID-19 questions.

**Characteristic**	**Frequency** ** (Percentage %)**
**Gender**	
Male	1696 (30.4)
Female	3892 (69.6)
**District**	
Damascus	2345 (42.1)
Idlib	4 (0.1)
Deir ez-Zur	20 (0.4)
Homs	673 (12.1)
Tartus	442 (7.9)
Latakia	482 (8.6)
Al Hasakah	58 (1.0)
Quneitra	13 (0.2)
Rif-Dimashq	661 (11.9)
Daraa	114 (2.0)
As Suwayda	203 (3.6)
Hama	303 (5.4)
Ar Raqqah	11 (0.2)
Aleppo	246 (4.4)
**Change place of living due to war**	
No	3480 (63.4)
Within the same city	919 (16.7)
To another city	1092 (19.9)
**Distress from war noises**	
No	884 (15.9)
Yes	4679 (84.1)
**Being a student**	
No	2371 (42.6)
In a school	133 (2.4)
In a university of high institute	3059 (55.0)
**Education**	
Elementary	26 (0.5)
Until grade 9	125 (2.2)
High School	784 (14.0)
University, or high institute	4064 (72.7)
Master or higher	589 (10.5)
**House that you currently live in**	
Owned	3566 (64.1)
Rented or given by the government	1433 (25.8)
Living in friends\relatives house	560 (10.1)
**Consanguinity between parents**	
No	3902 (70.2)
Third degree relatives	901 (16.2)
Fourth degree relatives	324 (5.8)
More distant relatives	433 (7.8)
**Chronic Medical Condition**	
No	1602 (66.4)
Hypertension	161 (6.7)
Diabetes	36 (1.5)
Asthma	176 (7.3)
Other	337 (14.0)
Two or more	101 (4.2)
**Chronic Condition With Housemates**	
No	1027 (25.8)
Hypertension	977 (24.5)
Diabetes	251 (6.3)
Asthma	154 (3.9)
Other	408 (10.3)
Two or more	1163 (29.2)
**Marital Status**	
Single	3271 (58.9)
In a relationship	477 (8.6)
Married	1426 (25.7)
Divorced	72 (1.3)
Engaged	274 (4.9)
Widowed	38 (0.7)
**Smoking cigarette or shisha**	
I was not a smoker and did not start	3609 (66.6)
I used to smoke one of them but now I smoke both	73 (1.3)
Yes I am smoking now and before COVID 19	1656 (30.5)
I started smoking cigarettes	32 (0.6)
I started Shisha	52 (1.0)
**Smoking Changes From COVID-19**	
Stopped smoking	94 (6.6)
Almost the same	460 (32.4)
Increased dramatically	322 (22.7)
Decreased dramatically	544 (38.3)
**Monthly Income adequacy**	
It cannot cover buying the essentials	1296 (23.3)
It is only enough for essentials from food and drink	3243 (58.3)
It is enough for essentials and other things	1023 (18.4)
**Have you been suspected of having COVID-19**	
No symptoms or signs	5445 (97.9)
Yes, has some possible symptoms, but no diagnosis	115 (2.1)
Yes, I was diagnosed with COVID-19 by a doctor	1 (>0.1)
**Exposed to someone likely to have COVID-19**	5306 (95.9)
No	
Yes, has some possible symptoms, but no diagnosis by doctor	214 (3.9)
Yes, with someone was diagnosed by a doctor	10 (0.2)
**Anyone in your family been diagnosed with COVID-19**	
No	5528 (99.2)
Yes, non-household member	23 (0.4)
Yes, member of household	23 (0.4)
**Happened because of COVID-19:**	
**Fallen ill physically**	
No	4883 (87.6)
To a family member	417 (7.5)
To a housemate	40 (0.7)
To you	237 (4.2)
**Hospitalized**	
No	5476 (98.2)
To a family member	73 (1.3)
To a housemate	12 (0.2)
To you	16 (0.3)
**Self-quarantine with symptoms**	
No	5549 (99.5)
To a family member	10 (0.2)
To a housemate	10 (0.2)
To you	8 (0.1)
**Self-quarantine without symptoms**	
No	5535 (99.2)
To a family member	16 (0.3)
To a housemate	11 (0.2)
To you	15 (0.3)
**Lost Job**	
No	3853 (69.7)
To a family member	822 (14.9)
To a housemate	78 (1.4)
To you	338 (6.1)
Two of the previous	374 (6.8)
All of the previous	63 (1.1)
**Reduced ability to earn money**	
No	3539 (64.0)
To a family member	973 (17.6)
To a housemate	107 (1.9)
To you	348 (6.3)
Two of the previous	473 (8.6)
All of the previous	88 (1.6)
**Passed away**	
No	5535 (99.3)
To a family member	32 (0.6)
To a housemate	6 (0.1)
**How worried have you been from:**	
**Being infected**	
Not at all	1255 (23.3)
Slightly	2262 (42.0)
Moderately	1170 (21.7)
Very	701 (13.0)
**Friends or family being infected**	
Not at all	318 (6.0)
Slightly	1472 (27.7)
Moderately	1476 (27.8)
Very	2045 (38.5)
**Your work will be negatively affected**	
Not at all	1821 (37.5)
Slightly	1026 (21.2)
Moderately	792 (16.3)
Very	1211 (25.0)
**Your studies will be negatively affected**	
Not at all	1749 (37.9)
Slightly	868 (18.8)
Moderately	846 (18.3)
Very	1153 (25.0)
**Providing food will be affected**	
Not at all	1043 (20.3)
Slightly	1592 (30.9)
Moderately	1225 (23.8)
Very	1290 (25.0)
**Are you committed to quarantine**	
I do not go out at all	1592 (28.6)
Only for essentials	3248 (58.3)
Reduced my going out	662 (11.9)
Have not changed at all	67 (1.2)
**Quality of your relationships with your friends**	
A lot better	78 (1.4)
A little better	368 (6.7)
About the same	3901 (70.7)
A little worse	841 (15.2)
A lot worse	330 (6.0)
**Quality of your relationships with your housemates**	
A lot better	302 (5.5)
A little better	1154 (21.0)
About the same	3067 (55.9)
A little worse	788 (14.4)
A lot worse	177 (3.2)
**Quality of the relationships between you and members of your family**	
A lot better	143 (2.6)
A little better	640 (11.7)
About the same	3867 (70.7)
A little worse	622 (11.4)
A lot worse	196 (3.6)
**Quality of your relationships with your Partner**	
A lot better	196 (6.7)
A little better	403 (13.8)
About the same	1658 (56.7)
A little worse	436 (14.9)
A lot worse	230 (7.9)
**Drugs increases after COVID-19**	
No drugs	3856 (71.6)
Analgesics and NSAIDS	1028 (19.1)
OTC flu medications	197 (3.7)
Hypnotics	32 (0.6)
Antibiotics	58 (1.1)
Other	128 (2.4)
2 drugs	72 (1.3)
3 drugs or more	14 (0.3)
**Drugs increases for your family**	
No drugs	3926 (73.3)
Analgesics and NSAIDS	839 (15.7)
OTC flu medications	237 (4.4)
Hypnotics	17 (0.3)
Antibiotics	85 (1.6)
Other	149 (2.8)
2 drugs	83 (1.5)
3 drugs or more	20 (0.4)

### Other Questions

Basic demographic questions were included on gender, age, educational level, whether they were currently a student, the governorate where they lived, and whether they had consanguineous parents. We asked whether they were distressed from war noises or having to change their place of living due to war and the number of these changes experienced.

### Definitions

Consanguinity was defined as third-degree consanguinity when the parents were first cousins, and fourth-degree consanguinity when the parents were second cousins, or second cousins once removed. We defined IT work type as engineering around a computer, IT, web design, or communication engineering that required programming. We defined engineering work types as civil, electrical/mechanical engineering, and architecture.

We defined a retail worker as a job selling costumers products, either in stores, or being a salesman. Medical engineering was part of “other health workers.” Working in television, radio, entertainment, or journalism were categorized in the “media” work type. We defined “office” work type as any work in an office either in a company or private practice such as accounting and law. A photographer or any job involving music, painting, or design except for web design was included in the “Art and music” category.

### Data Process

Data were processed using IBM SPSS software version 26 for Windows (SPSS Inc., IL, USA). Chi-square, one-way analysis of variance (ANOVA), linear regression, and independent *t*-tests were performed to determine the statistical significance between the groups. Pearson correlation was also calculated. Through the same software, odds ratios (ORs) and the 95% confidence intervals for the groups were calculated using Mantel–Haenszel test. Values of <0.05 for the two-tailed *P* values were considered statistically significant.

## Results

**Figure 1:**
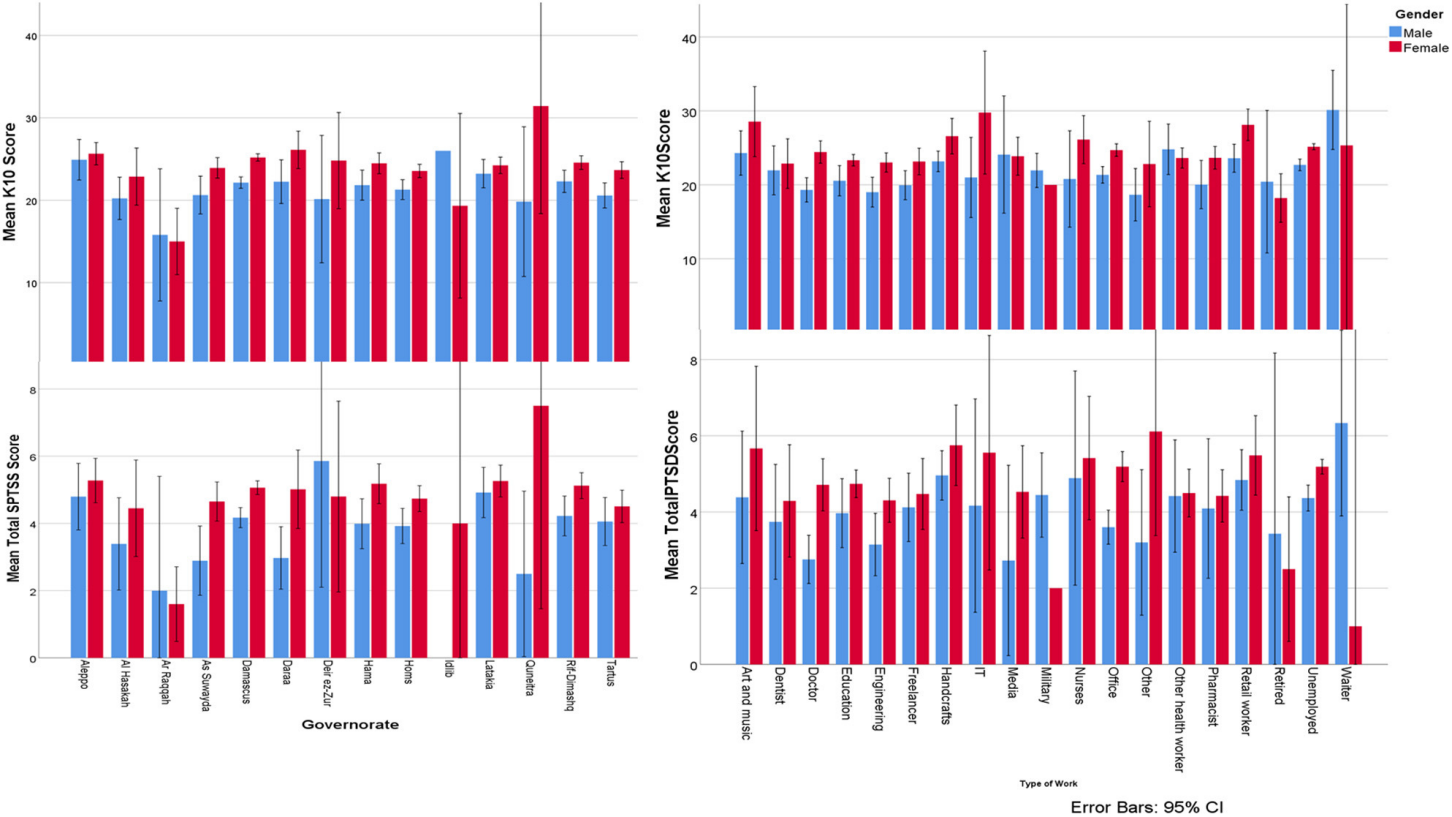
SPTSS and K10 scores by gender distribution according to the type of work and governorates.

Our sample comprised 5,588 participants from all across Syria with 3,892 (69.6%) female participants. The mean age was 26.84 ± 7.815 years. In the sample, 37.8% were well according to K10, but 27.6% had a probable severe mental disorder. Approximately, 37% did not report positive SPTSS items, and 23.3% met the criteria for probable PTSD. Characteristics of subjects, their responses to COVID-19 questions, war variables, and other nominal variables are demonstrated in Table 1. Age, PTSD, MSPSS, K10 scores, and results on other war and numeral variables are demonstrated in Table 2. COVID-19 questions, war, and other variables associated with SPTSS items and total scores are demonstrated in Table 3 with each social support and total MSPSS support scores demonstrated in Table 4, and K10 + LM scores and days demonstrated in Table 5. PTSD, and K10 scores distributions in governorates by gender and according to the type of work are demonstrated in Figure 1. The associations between PTSD clusters, K10 scores, and social support are demonstrated in Table 6.

**Table 2:** Age, PTSD, social support, and K10 results and scores with other numeric variables.

**Characteristic**	**Frequency** ** (Percentage %)**	**Characteristic**	**Mean (Std. Deviation)**
**Positive Avoidance Prevalence**	1891 (35.6)	**Age**	26.84 (7.815)
**Positive Arousal Prevalence**	2558 (48.7)	**Changing place of living due to war**	0.45 (0.752)
**Positive Re-experience Prevalence**	2305 (44.3)	**Number of people living in the house**	2.59 (0.605)
**Positive PTSD Number prevalence**		**How many rooms of your current house**	3.837 (1.6269)
No PTSD	1909 (36.8)	**Number of children under 18 living in the house**	1.22 (1.666)
One positive subscale	1061 (20.5)	**Avoidance score**	1.98 (1.748)
Two positive subscales	1005 (19.4)	**Arousal score**	1.70 (1.504)
Three positive subscales	1209 (23.3)	**Re-experience score**	1.03 (1.458)
**Significant Other Support**		**Total PTSD score**	4.74 (4.051)
Low Support	1038 (19.3)	**Significant other score**	19.257 (7.3549)
Moderate Support	1365 (25.4)	**Family score**	19.155 (6.5910)
High Support	2968 (55.3)	**Friends score**	16.082 (7.2203)
**Family Support**		**Total support score**	54.46 (17.041)
Low Support	908 (16.9)	**K10 score**	23.8473 (9.10760)
Moderate Support	1590 (29.6)	**Days unable to carry normal activities and work due to negative feelings**	6.91 (7.662)
High Support	2865 (53.4)	**Days had to reduce normal activities and work due to negative feelings**	9.53 (8.903)
**Friends Support**			
Low Support	1690 (31.7)		
Moderate Support	1697 (31.8)		
High Support	1950 (36.5)		
**Total Support**			
Low Support	796 (14.9)		
Moderate Support	2449 (45.9)		
High Support	2087 (39.1)		
**K10Result**			
Well	2039 (37.8)		
Mild Mental Disorder	1056 (19.6)		
Moderate Mental Disorder	812 (15.0)		
Severe Mental Disorder	1489 (27.6)		

**Table 3:** SPTSS items and total mean scores and correlations with COVID-19 and war variables along with other variables.

**Characteristic**	**Avoidance**			**Arousal**			**Re-experience**			**Total SPSS Score**		
	**Mean**	**Std.** ** Dev**.	**P value**	**Mean**	**Std.** ** Dev**.	**P value**	**Mean**	**Std.** ** Dev**.	**P value**	**Mean**	**Std.** ** Dev**.	**P value**
**Gender**												
Male	1.8	1.8	<0.001	1.5	1.4	<0.001	0.8	1.3	<0.001	4.1	3.9	<0.001
Female	2.1	1.7		1.8	1.5		1.1	1.5		5	4.1	
**Change place of living due to war**												
No	1.9	1.7	<0.001	1.6	1.5	<0.001	1	1.4	0.001	4.5	4	<0.001
Within the same city	2.1	1.8		1.8	1.5		1.1	1.5		5	4.1	
To another city	2.2	1.8		1.9	1.5		1.2	1.5		5.2	4.1	
**Distress from war noises**												
No	1.8	1.7	0.002	1.4	1.4	<0.001	0.8	1.3	<0.001	4.1	3.8	<0.001
Yes	2	1.7		1.8	1.5		1.1	1.5		4.9	4.1	
**Being a student**												
No	1.8	1.7	<0.001	1.6	1.5	<0.001	0.9	1.4	<0.001	4.4	4	<0.001
In a school	2.2	1.6		1.7	1.5		1.3	1.5		5.1	3.9	
In a university of high institute	2.1	1.8		1.8	1.5		1.1	1.5		5	4.1	
**Education**												
Elementary	1.5	1.8	0.006	1.4	1.6	<0.001	0.9	1.5	0.002	4	4.6	<0.001
Until grade 9	1.9	1.7		1.5	1.5		1.2	1.4		4.7	4	
High School	2.1	1.7		1.7	1.5		1.1	1.5		5	4	
University, or high institute	2	1.8		1.7	1.5		1	1.5		4.8	4.1	
Master or higher	1.8	1.7		1.4	1.4		0.8	1.3		4	3.8	
**House that you currently live in**												
Owned	1.9	1.7	<0.001	1.6	1.5	0.001	1	1.5	0.089	4.6	4	0.001
Rented or given by the government	2.1	1.8		1.8	1.5		1.1	1.5		5	4.1	
Living in friends\relatives house	2.1	1.8		1.8	1.5		1.1	1.5		5.1	4	
**Chronic Medical Condition**												
No	1.9	1.7	<0.001	1.6	1.5	<0.001	0.9	1.4	<0.001	4.4	3.9	<0.001
Hypertension	1.9	1.9		1.6	1.6		1.1	1.6		4.7	4.5	
Diabetes	1.5	1.8		1.6	1.6		1	1.4		4.4	4.3	
Asthma	2.6	1.8		2.2	1.6		1.5	1.6		6.3	4.3	
Other	2.5	1.8		2.2	1.6		1.5	1.6		6.2	4.3	
Two or more	2.3	1.8		2	1.6		1.4	1.7		5.8	4.4	
**Chronic Condition With Housemates**												
No	1.8	1.7	<0.001	1.6	1.5	<0.001	0.9	1.4	<0.001	4.3	3.8	<0.001
Hypertension	2	1.7		1.6	1.5		1	1.4		4.6	4	
Diabetes	1.9	1.7		1.6	1.5		0.9	1.4		4.4	3.9	
Asthma	2.2	1.8		1.9	1.5		1.5	1.6		5.7	4.1	
Other	2.2	1.9		2	1.6		1.3	1.6		5.6	4.4	
Two or more	2.2	1.8		1.9	1.5		1.1	1.5		5.2	4.2	
**Marital Status**												
Single	2.1	1.7	<0.001	1.7	1.5	0.002	1.1	1.5	0.009	4.8	4	<0.001
In a relationship	2.1	1.8		2	1.5		1.2	1.5		5.2	4.1	
Married	1.7	1.7		1.6	1.5		0.9	1.4		4.3	3.9	
Divorced	1.8	1.9		1.5	1.4		1.1	1.5		4.3	4.3	
Engaged	2.2	1.9		1.7	1.5		1.1	1.6		5	4.3	
Widowed	2.6	2.2		1.8	1.5		1.4	1.5		5.8	4.5	
**Smoking cigarette or shisha**												
I was not a smoker and did not start	2	1.7	0.001	1.7	1.5	0.001	1	1.4	<0.001	4.7	3.9	<0.001
I used to smoke one of them but now I smoke both	2.8	2		2.4	1.6		1.7	1.7		6.9	4.5	
Yes I am smoking now and before COVID 19	2	1.8		1.7	1.5		1.1	1.5		4.8	4.2	
I started smoking cigarettes	2.2	2.3		1.3	1.5		1	1.5		4.6	5	
I started Shisha	2.4	2.1		1.8	1.7		1.4	1.8		5.6	5	
**Smoking Changes From COVID-19**												
Stopped smoking	1.9	1.6	<0.001	1.7	1.5	<0.001	0.8	1.3	<0.001	4.5	4	<0.001
Almost the same	1.8	1.7		1.5	1.5		0.9	1.4		4.2	4	
Increased dramatically	2.5	2		2.2	1.6		1.5	1.7		6.2	4.6	
Decreased dramatically	2	1.7		1.7	1.5		1	1.4		4.8	3.9	
**Monthly Income adequacy**												
It cannot cover buying the essentials	2.3	1.8	<0.001	1.9	1.6	<0.001	1.3	1.6	<0.001	5.6	4.3	<0.001
It is only enough for essentials from food and drink	1.9	1.7		1.7	1.5		1	1.4		4.6	3.9	
It is enough for essentials and other things	1.7	1.7		1.5	1.5		0.9	1.4		4.1	3.9	
**Have you been suspected of having COVID-19**												
No symptoms or signs	2	1.7	0.005	1.7	1.5	0.002	1	1.5	0.034	4.7	4	0.001
Yes, has some possible symptoms, but no diagnosis	2.5	1.9		2.2	1.6		1.4	1.7		6.2	4.4	
Yes, I was diagnosed with COVID-19 by a doctor	1	.		3	.		1	.		5	.	
**Exposed to someone likely to have COVID-19**												
No	2	1.7	<0.001	1.7	1.5	<0.001	1	1.4	<0.001	4.7	4	<0.001
Yes, has some possible symptoms, but no diagnosis by doctor	2.5	1.8		2.2	1.6		1.3	1.6		6	4.3	
Yes, with someone was diagnosed by a doctor	3.3	2.2		2.5	1.4		2.4	2		8.2	4.2	
**Anyone in your family been diagnosed with COVID-19**												
No	2	1.7	0.339	1.7	1.5	0.252	1	1.5	0.111	4.7	4	0.141
Yes, non-household member	2.4	1.9		2.2	1.5		1.7	1.9		6.4	4.9	
Yes, member of household	2.3	1.6		1.7	1.5		1.1	1.3		5.1	3.3	
**Happened because of COVID-19:**												
**Fallen ill physically**												
No	1.9	1.7	<0.001	1.6	1.5	<0.001	1	1.4	<0.001	4.5	4	<0.001
To a family member	2.4	1.9		2.1	1.5		1.4	1.6		6	4.3	
To a housemate	2.4	1.9		2	1.7		1.3	1.6		5.8	4.4	
To you	2.5	1.8		2.2	1.6		1.6	1.7		6.5	4.3	
**Hospitalized**												
No	2	1.7	0.117	1.7	1.5	0.274	1	1.5	0.053	4.7	4	0.064
To a family member	2.4	2		2	1.6		1.4	1.7		5.8	4.7	
To a housemate	2.6	2		2	1.8		1.6	1.9		6.2	5.1	
To you	2.3	2.3		1.7	1.4		1.4	1.7		5.5	4.7	
**Self-quarantine with symptoms**												
No	2	1.7	<0.001	1.7	1.5	0.025	1	1.5	0.001	4.7	4	<0.001
To a family member	4	2.2		3	1.4		2.7	1.3		9.7	4.6	
To a housemate	3.2	1.9		2.1	1.7		1.9	1.8		7.3	4.9	
To you	3.3	2.1		2.3	1.6		1.6	2.1		7.1	4.6	
**Self-quarantine without symptoms**												
No	2	1.7	0.013	1.7	1.5	0.025	1	1.5	0.001	4.7	4	0.004
To a family member	2.6	1.8		2.7	1.3		1.5	1.6		6.8	4.4	
To a housemate	3.5	1.8		1.9	1.7		2	1.7		7.4	4.3	
To you	2.5	1.4		2.4	1.8		2.4	1.8		7.3	4.5	
**Lost Job**												
No	1.8	1.7	<0.001	1.6	1.5	<0.001	1	1.4	<0.001	4.4	4	<0.001
To a family member	2.2	1.7		1.9	1.5		1.2	1.5		5.4	4	
To a housemate	2	1.7		2.1	1.6		1.5	1.7		5.8	4.4	
To you	2.1	1.8		1.9	1.5		1	1.4		5.1	4.1	
Two of the previous	2.4	1.7		1.8	1.5		1.1	1.5		5.4	3.9	
All of the previous	2.9	1.7		2.1	1.6		1.5	1.7		6.4	4.5	
**Reduced ability to earn money**												
No	1.8	1.7	<0.001	1.5	1.5	<0.001	0.9	1.4	<0.001	4.3	4	<0.001
To a family member	2.3	1.7		2	1.5		1.2	1.5		5.4	4	
To a housemate	2.1	1.8		2.2	1.6		1.5	1.6		5.9	4.4	
To you	2.1	1.8		1.9	1.6		1.1	1.4		5.1	4.1	
Two of the previous	2.4	1.7		1.9	1.5		1.2	1.5		5.5	4	
All of the previous	2.7	1.7		2.2	1.6		1.6	1.7		6.4	4.4	
**Passed away**												
No	2	1.7	<0.001	1.7	1.5	<0.001	1	1.5	<0.001	4.7	4	<0.001
To a family member	3.3	2		2.9	1.6		2.1	1.9		8.3	4.9	
To a housemate	3.8	1.7		2.2	1.9		2	2.1		8	5.1	
**How worried have you been from:**												
**Being infected**												
Not at all	1.8	1.8	<0.001	1.4	1.4	<0.001	0.9	1.4	<0.001	4.1	4	<0.001
Slightly	1.8	1.6		1.5	1.4		0.9	1.3		4.2	3.7	
Moderately	2.1	1.7		1.8	1.5		1.1	1.4		5	3.9	
Very	2.9	1.8		2.6	1.5		1.8	1.8		7.3	4.3	
**Friends or family being infected**												
Not at all	1.5	1.8	<0.001	1.3	1.3	<0.001	0.7	1.3	<0.001	3.5	3.8	<0.001
Slightly	1.7	1.7		1.3	1.4		0.8	1.3		3.8	3.7	
Moderately	1.9	1.7		1.6	1.4		0.9	1.4		4.5	3.8	
Very	2.4	1.8		2.1	1.5		1.4	1.6		5.9	4.2	
**Your work will be negatively affected**												
Not at all	1.7	1.7	<0.001	1.5	1.4	<0.001	0.9	1.4	<0.001	4.1	3.8	<0.001
Slightly	1.8	1.7		1.5	1.4		0.9	1.4		4.3	3.8	
Moderately	2	1.8		1.6	1.4		0.9	1.4		4.5	3.9	
Very	2.5	1.8		2.1	1.6		1.4	1.6		6.1	4.4	
**Your studies will be negatively affected**												
Not at all	1.8	1.7	<0.001	1.6	1.5	<0.001	0.9	1.4	<0.001	4.3	3.8	<0.001
Slightly	1.8	1.6		1.5	1.4		0.9	1.4		4.1	3.8	
Moderately	2	1.7		1.6	1.5		1	1.4		4.6	3.9	
Very	2.6	1.8		2.2	1.6		1.5	1.6		6.2	4.3	
**Providing food will be affected**												
Not at all	1.5	1.6	<0.001	1.3	1.4	<0.001	0.8	1.3	<0.001	3.6	3.6	<0.001
Slightly	1.7	1.6		1.5	1.4		0.8	1.3		4	3.6	
Moderately	2.1	1.8		1.7	1.5		1	1.4		4.9	3.9	
Very	2.7	1.8		2.3	1.6		1.6	1.7		6.5	4.3	
**Are you committed to quarantine**												
I do not go out at all	2.2	1.8	<0.001	1.8	1.5	<0.001	1.2	1.5	<0.001	5.2	4.2	<0.001
Only for essentials	1.9	1.7		1.7	1.5		1	1.4		4.6	4	
Reduced my going out	1.8	1.7		1.6	1.5		1	1.4		4.4	4	
Have not changed at all	2	2		1.7	1.6		1.1	1.5		4.9	4.4	
**Quality of your relationships with your friends**												
A lot better	1.7	1.4	<0.001	1.6	1.5	<0.001	0.8	1.4	<0.001	4	3.4	<0.001
A little better	2	1.7		1.8	1.6		1.1	1.5		4.8	4.2	
About the same	1.8	1.7		1.6	1.5		0.9	1.4		4.3	3.8	
A little worse	2.5	1.8		2.1	1.6		1.4	1.6		6.1	4.3	
A lot worse	2.8	1.9		2.2	1.6		1.7	1.8		6.7	4.6	
**Quality of your relationships with your housemates**												
A lot better	1.6	1.5	<0.001	1.5	1.4	<0.001	0.8	1.3	<0.001	3.9	3.5	<0.001
A little better	1.9	1.7		1.6	1.5		1	1.4		4.6	3.9	
About the same	1.8	1.7		1.5	1.5		0.9	1.3		4.2	3.9	
A little worse	2.7	1.7		2.4	1.5		1.5	1.6		6.6	4.1	
A lot worse	3.5	1.7		3.1	1.4		2.3	1.8		8.9	4.3	
**Quality of the relationships between you and members of your family**												
A lot better	1.5	1.4	<0.001	1.5	1.4	<0.001	0.8	1.2	<0.001	3.8	3.3	<0.001
A little better	1.9	1.7		1.6	1.4		1	1.4		4.5	3.8	
About the same	1.9	1.7		1.6	1.5		0.9	1.4		4.4	3.9	
A little worse	2.6	1.8		2.2	1.5		1.5	1.7		6.3	4.2	
A lot worse	3	1.8		2.6	1.7		2	1.8		7.6	4.6	
**Quality of your relationships with your Partner**												
A lot better	1.6	1.7	<0.001	1.6	1.5	<0.001	0.9	1.4	<0.001	4.1	3.9	<0.001
A little better	1.9	1.7		1.8	1.5		1.1	1.5		4.8	4.1	
About the same	1.6	1.7		1.5	1.5		0.8	1.3		4	3.8	
A little worse	2.6	1.8		2.2	1.5		1.4	1.6		6.2	4.1	
A lot worse	3.3	1.8		2.8	1.6		2.3	1.8		8.5	4.4	
**Drugs increases after COVID-19**												
No drugs	1.8	1.7	<0.001	1.5	1.4	<0.001	0.8	1.3	<0.001	4.2	3.8	<0.001
Analgesics and NSAIDS	2.4	1.9		2.1	1.6		1.5	1.7		6	4.4	
OTC flu medications	1.9	1.7		1.8	1.5		1.2	1.5		4.9	4.1	
Hypnotics	3.3	1.7		3.1	1.5		2.7	1.9		9.2	4	
Antibiotics	2.3	1.6		2.1	1.5		1.3	1.7		5.8	4	
Other	2.3	2		2.1	1.5		1.2	1.5		5.6	4.2	
2 drugs	2.4	1.8		2.2	1.6		1.7	1.7		6.3	4.4	
3 drugs or more	1.9	2		2.5	1.7		1.5	1.8		5.9	5.1	
**Drugs increases for your family**												
No drugs	1.9	1.7	<0.001	1.6	1.5	<0.001	0.9	1.4	<0.001	4.4	3.9	<0.001
Analgesics and NSAIDS	2.3	1.8		2	1.6		1.3	1.6		5.6	4.2	
OTC flu medications	2	1.7		1.8	1.4		1	1.4		4.9	3.9	
Hypnotics	2.4	1.4		2.5	1.3		1.5	1.3		6.5	2.9	
Antibiotics	2.7	1.7		2.4	1.5		1.8	1.7		6.8	4.2	
Other	2.2	1.9		2	1.5		1.3	1.5		5.5	4.1	
2 drugs	2.1	1.6		1.8	1.5		1.2	1.4		5.1	4.1	
3 drugs or more	2.5	1.3		2.3	1.8		1.5	1.9		6.3	4	

**Table 4:** MSPSS items and total scores mean scores and correlations with COVID-19 and war variables along with other variables.

**Characteristic**	**Significant Other Support**			**Family Support**			**Friends Support**			**Total MSPSS Score**		
	**Mean**	**Std.** ** Dev**.	**P value**	**Mean**	**Std.** ** Dev**.	**P value**	**Mean**	**Std.** ** Dev**.	**P value**	**Mean**	**Std.** ** Dev**.	**P value**
**Gender**												
Male	18.4	7.6	<0.001	18.7	6.6	0.002	16.3	7.1	0.096	53.4	17.7	0.004
Female	19.6	7.2		19.3	6.6		16	7.3		54.9	16.7	
**Change place of living due to war**												
No	19.4	7.4	0.04	19.4	6.6	<0.001	16.3	7.2	0.023	55.1	17.1	0.001
Within the same city	18.8	7.2		18.7	6.5		15.9	7.2		53.3	16.8	
To another city	19.2	7.4		18.6	6.6		15.6	7.2		53.4	17	
**Distress from war noises**												
No	18.3	7.6	<0.001	18.2	6.9	<0.001	15.8	7.4	0.227	52.3	17.8	<0.001
Yes	19.4	7.3		19.3	6.5		16.1	7.2		54.9	16.9	
**Being a student**												
No	20.3	7	<0.001	19.8	6.5	<0.001	16.2	7.2	0.459	56.3	16.9	<0.001
In a school	18.2	7.5		18.6	6.7		15.6	7.8		52.2	17.8	
In a university of high institute	18.5	7.5		18.7	6.6		16	7.2		53.2	17	
**Education**												
Elementary	22.7	5.6	<0.001	18.7	6.6	<0.001	14.7	7.7	<0.001	56.1	16.5	<0.001
Until grade 9	18.2	7.6		17.7	7.1		13.9	7.7		49.7	18.2	
High School	18.4	7.3		18.2	6.7		15.6	7.3		52.2	17.4	
University, or high institute	19.3	7.3		19.1	6.6		16.1	7.2		54.4	17	
Master or higher	20.4	7.3		20.9	6.2		17.3	7		58.7	16.1	
**House that you currently live in**												
Owned	19.4	7.3	0.184	19.5	6.5	<0.001	16.3	7.1	0.004	55.1	17	0.001
Rented or given by the government	19.2	7.3		18.7	6.7		15.6	7.3		53.5	17.2	
Living in friends\relatives house	18.8	7.6		18.3	6.6		15.6	7.3		52.8	16.8	
**Chronic Medical Condition**												
No	20.6	7.4	0.001	20.3	6.5	<0.001	17	7.2	0.002	57.9	16.8	<0.001
Hypertension	19.2	7.3		18.4	6.8		16.1	7.2		53.7	17.8	
Diabetes	20.1	8.1		19.5	7		14.9	8		54.4	19.8	
Asthma	18.8	8.2		18.9	7.3		15.1	7.7		52.8	18.9	
Other	19.1	7.8		18.2	7		15.9	7.4		53.2	17.6	
Two or more	18.7	7.9		17.6	6.9		15.7	7.4		52.1	19.1	
**Chronic Condition With Housemates**												
No	20.9	7.3	<0.001	20.4	6.7	<0.001	16.8	7.3	0.204	58	16.8	<0.001
Hypertension	18.8	7.3		19	6.4		16.3	7.1		54.1	16.6	
Diabetes	19.9	7.5		18.9	7		16.7	7.7		55.4	18.6	
Asthma	19	7.7		18.6	7.1		15.8	7.2		53.3	17.9	
Other	19.5	7.4		18.8	6.6		15.8	7.4		54	17.4	
Two or more	18.9	7.5		18.7	6.7		16.2	7.2		53.8	17	
**Marital Status**												
Single	17.3	7.4	<0.001	18.7	6.6	<0.001	16.2	7.2	0.611	52.1	17.3	<0.001
In a relationship	23.2	5.4		18.4	6.6		16.4	7.3		57.9	14.9	
Married	21.8	6.3		20.5	6.3		15.8	7.2		58	16.5	
Divorced	18.3	7.8		18.1	7		16	7.5		52	18.2	
Engaged	23.7	5.4		19.9	6.4		16.1	7.4		59.7	14.9	
Widowed	17.8	7.3		17.9	6		14.9	6.4		50	15.2	
**Smoking cigarette or shisha**												
I was not a smoker and did not start	19.3	7.3	0.413	19.4	6.5	0.001	16.1	7.2	0.211	54.7	16.9	0.049
I used to smoke one of them but now I smoke both	17.6	7.8		17.1	7.1		14.2	7.3		48.9	16.6	
Yes I am smoking now and before COVID 19	19.3	7.4		18.9	6.7		16.2	7.2		54.4	17.4	
I started smoking cigarettes	19.5	7.5		18.5	7.6		16.7	7.4		54.6	17.2	
I started Shisha	18.8	7.8		17.1	7		15.6	7.2		51.6	17.8	
**SmokingChangesFromCOVID19**												
Stopped smoking	20.2	6.9	0.049	19.8	6.2	0.001	17.1	7.3	0.001	57.1	16.5	<0.001
Almost the same	19.8	7.2		19.5	6.5		16.2	7.2		55.4	16.9	
Increased dramatically	18.4	7.5		17.6	7		14.8	7.1		50.7	17.5	
Decreased dramatically	19.5	7.3		18.8	6.8		16.9	7.2		55.2	17.3	
**Monthly Income Adequacy**												
It cannot cover buying the essentials	18.6	7.8	<0.001	17.9	7.2	<0.001	14.6	7.3	<0.001	51	18.1	<0.001
It is only enough for essentials from food and drink	19.2	7.2		19.3	6.4		16.3	7.1		54.8	16.6	
It is enough for essentials and other things	20.2	7.1		20.3	6.2		17.3	7.2		57.8	16.4	
**Have you been suspected of having COVID-19**												
No symptoms or signs	19.3	7.3	0.381	19.2	6.6	0.002	16.1	7.2	0.059	54.6	17	0.014
Yes, has some possible symptoms, but no diagnosis	18.4	8		17	6.9		14.9	7.4		50	18.2	
Yes, I was diagnosed with COVID-19 by a doctor	14.5	.		19	.		4	.		37.5	.	
**Exposed to someone likely to have COVID-19**												
No	19.3	7.3	0.075	19.2	6.6	0.354	16.1	7.2	0.751	54.6	17	0.17
Yes, has some possible symptoms, but no diagnosis by doctor	18.2	7.8		18.5	6.6		15.8	7		52.4	17	
Yes, with someone was diagnosed by a doctor	17.3	7.1		18.7	7.9		15.3	8.6		51.3	22	
**Anyone in your family been diagnosed with COVID-19**												
No	19.3	7.3	0.03	19.2	6.6	0.026	16.1	7.2	0.023	54.5	17	0.005
Yes, non-household member	17.6	7.6		18.9	6.8		15.4	6.8		52.4	15.9	
Yes, member of household	15.5	7.5		15.4	7.3		11.9	6.8		42.8	18.4	
**Happened because of COVID-19:**												
**Fallen ill physically**												
No	19.4	7.3	<0.001	19.3	6.5	<0.001	16.2	7.2	0.007	54.9	17	<0.001
To a family member	18.2	7.5		17.8	7		15.2	7.3		51.2	17.3	
To a housemate	18.6	6.9		17.2	7.9		15.1	6.6		51.1	18.2	
To you	17.9	7.7		18.1	6.7		15.1	7.4		51	17.2	
**Hospitalized**												
No	19.3	7.4	0.933	19.2	6.6	0.134	16.1	7.2	0.582	54.5	17	0.41
To a family member	19	7.6		17.6	6.9		14.9	7.4		51.3	17.8	
To a housemate	18.8	7.6		17.3	8.9		15.3	6.7		51.3	22.2	
To you	20.2	7.6		17.9	8.6		16.1	6.3		54.2	17.8	
**Self-quarantine with symptoms**												
No	19.3	7.4	0.06	19.2	6.6	<0.001	16.1	7.2	0.071	54.5	17	0.002
To a family member	19.8	6.7		21.1	6.9		16.3	7.5		57.2	17.9	
To a housemate	13.5	6.9		10	8.1		11.5	5.8		35	19.9	
To you	22	6.1		19	6.9		20.5	5.3		61.5	14.8	
**Self-quarantine without symptoms**												
No	19.3	7.3	0.117	19.2	6.6	<0.001	16.1	7.2	0.031	54.5	17	0.001
To a family member	18.4	7.6		16.2	7.1		14.8	6.5		49.4	16.2	
To a housemate	14.1	7.6		10	8.2		10	4.4		34.1	18.7	
To you	20.2	8		19.3	6.8		17.4	7.5		56.9	18.4	
**Lost Job**												
No	19.4	7.3	0.03	19.5	6.6	<0.001	16.4	7.2	<0.001	55.3	17	<0.001
To a family member	18.6	7.6		18.7	6.3		15.5	7.2		52.7	17	
To a housemate	20.5	6.4		18.6	6.5		13.8	7.2		52.9	15.6	
To you	18.8	7.5		18	6.9		15.6	7.4		52.5	17.4	
Two of the previous	19.2	7.5		18.4	6.7		15.5	7.3		53.1	16.8	
All of the previous	19	7.2		18.1	6.9		14.4	7.1		51	18	
**Reduced ability to earn money**												
No	19.5	7.3	<0.001	19.6	6.5	<0.001	16.4	7.2	<0.001	55.6	17	<0.001
To a family member	18.6	7.5		18.6	6.5		15.7	7.2		52.9	17	
To a housemate	20.5	6.8		19.4	6.6		14.9	7.6		54.7	17.2	
To you	18.4	7.7		18.1	6.8		15.2	7.2		51.7	17.6	
Two of the previous	18.7	7.4		18.2	6.6		15.6	7.1		52.4	16.5	
All of the previous	19.7	7.2		17.9	6.6		14.1	7.4		51.5	17.9	
**Passed away**												
No	19.3	7.3	0.02	19.2	6.6	0.002	16.1	7.2	0.007	54.5	17	<0.001
To a family member	16.6	8.5		17.4	7.1		13.5	6.8		47.5	19.1	
To a housemate	13	6.2		11	8		10	5.2		34	18	
**How worried have you been from:**												
**Being infected**												
Not at all	19	7.7	0.328	18.8	6.8	0.016	15.9	7.6	<0.001	53.7	18.1	0.009
Slightly	19.3	7.2		19.3	6.4		16.3	7		54.9	16.7	
Moderately	19.5	7.2		19.5	6.4		16.6	7.2		55.4	16.8	
Very		19.1	7.3		18.7	7		15.2	7.2		53	16.3
**Friends or family being infected**												
Not at all	19.5	7.7	0.606	18.7	7.3	0.263	15.8	7.6	0.286	54.1	18.7	0.715
Slightly	19.5	7.1		19.1	6.6		16.4	7.1		55	17.1	
Moderately	19.2	7.4		19.1	6.4		16.2	7.2		54.4	16.9	
Very	19.2	7.4		19.4	6.6		16	7.2		54.6	16.6	
**Your work will be negatively affected**												
Not at all	19.7	7.4	0.034	19.6	6.6	<0.001	16.4	7.3	0.001	55.8	17.3	<0.001
Slightly	19.4	7.1		19.6	6.3		16.6	7.1		55.5	16.9	
Moderately	19.2	7.1		18.9	6.4		16.3	6.9		54.3	16.6	
Very	19	7.6		18.7	6.8		15.5	7.2		53.2	16.9	
**Your studies will be negatively affected**												
Not at all	19.9	7.5	<0.001	19.4	6.7	0.016	16.2	7.3	0.101	55.5	17.5	0.001
Slightly	19.2	7.1		19.2	6.3		16.5	7.1		54.9	16.5	
Moderately	18.6	7.4		19.1	6.5		16.4	7		53.9	16.9	
Very	18.4	7.5		18.6	6.7		15.8	7.4		52.8	16.8	
**Providing food will be affected**												
Not at all	20.2	7.3	<0.001	19.5	6.8	0.032	16.7	7.4	<0.001	56.4	17.5	<0.001
Slightly	19	7.1		19.3	6.3		16.2	7		54.6	16.7	
Moderately	19.2	7.4		19.2	6.5		16.3	7.2		54.7	17.1	
Very	18.9	7.6		18.7	6.8		15.5	7.2		53.1	16.8	
**Are you committed to quarantine**												
I do not go out at all	19	7.5	0.164	19.1	6.7	0.774	15.6	7.5	0.005	53.6	17.4	0.145
Only for essentials	19.5	7.3		19.2	6.5		16.2	7.1		54.8	16.8	
Reduced my going out	19.1	7.6		19.1	6.6		16.7	7.2		54.8	17.4	
Have not changed at all	18.8	6.9		18.4	7.1		16.4	7.2		53.8	17.3	
**Quality of your relationships with your friends**												
A lot better	22.8	6.4	<0.001	20.1	7.2	<0.001	21	6.1	<0.001	63.6	15.3	<0.001
A little better	19.4	7		19	6.5		17.3	6.7		55.6	16.3	
About the same	19.7	7.2		19.5	6.4		16.6	7.1		55.8	16.6	
A little worse	17.7	7.7		18.1	6.7		14.3	7		50.1	16.9	
A lot worse	17.5	8.2		17.4	7.6		12.4	7.4		47.4	18.7	
**Quality of your relationships with your housemates**												
A lot better	23	6.4	<0.001	23.5	5.1	<0.001	17.9	7.2	<0.001	64.5	14	<0.001
A little better	19.8	7		19.9	5.8		16.7	7		56.4	15.8	
About the same	19.4	7.3		19.6	6.4		16.3	7.2		55.3	16.8	
A little worse	17.5	7.5		16.2	6.6		14.7	7.2		48.4	16.6	
A lot worse	15	8.3		12.8	7.3		12.1	6.9		40	18	
**Quality of the relationships between you and members of your family**												
A lot better	22.9	6.8	<0.001	23.2	5.2	<0.001	17.6	7.7	<0.001	63.6	14.8	<0.001
A little better	19.9	6.8		19.8	6		16.3	7.1		56.1	15.8	
About the same	19.4	7.3		19.4	6.5		16.4	7.1		55.1	16.7	
A little worse	17.8	7.8		17.5	6.9		14.6	7.4		50	18.1	
A lot worse	16.6	8.2		15.9	7.8		13.6	7.5		46	18.5	
**Quality of your relationships with your Partner**												
A lot better	25.7	3.9	<0.001	22.3	5.9	<0.001	17.2	7.6	<0.001	65.3	13.1	<0.001
A little better	22.2	5.6		19.4	6		16.1	7.3		57.8	15.1	
About the same	21.6	6.5		20.1	6.4		16.7	7.2		58.4	16.3	
A little worse	19.6	7		17.8	6.5		14.5	7.3		51.9	16.4	
A lot worse	15.6	7.7		15.9	7.1		13.3	7.5		44.8	18.1	
**Drugs increases after COVID-19**												
No drugs	19.3	7.4	0.655	19.3	6.6	0.317	16.3	7.2	0.046	54.9	17.1	0.126
Analgesics and NSAIDS	19	7.1		18.8	6.4		15.5	7.2		53.2	16.6	
OTC flu medications	19.2	7.3		19.1	6.5		15.9	6.7		54	17.1	
Hypnotics	18.4	9		19.4	7.5		15.1	8.2		52.4	20.2	
Antibiotics	19.1	6.2		17.7	7		15.1	7.4		51.6	16.4	
Other	20.2	7.8		19.4	6.6		16.5	7.3		56.2	17.8	
2 drugs	20.1	7.6		18.9	7		15.7	7.4		54.9	17.3	
3 drugs or more	20.2	8.7		20.8	6.7		15.4	8.8		56.4	17.3	
**Drugs increases for your family**												
No drugs	19.3	7.4	0.079	19.3	6.6	0.047	16.3	7.2	0.148	54.9	17.1	0.029
Analgesics and NSAIDS	18.9	7.4		19	6.5		15.6	7.2		53.5	16.7	
OTC flu medications	19.7	6.4		18.5	6.3		16.2	7.2		54.3	16.3	
Hypnotics	20.7	6.4		17.6	7.9		15.9	6.4		54.2	18.1	
Antibiotics	18.2	6.8		18.2	6.7		15.3	7.5		51.5	16.8	
Other	20	7		19.4	6.4		16.5	7.2		55.9	16.4	
2 drugs	18.6	7.6		19.2	5.9		15	7.3		52.5	16.2	
3 drugs or more	15.6	6.9		15.4	6.2		13.8	4.7		44.7	13.2	

**Table 5:** K10 scores, days unable to work and days of reduced work and correlations with COVID-19 and war variables along with other variables.

**Characteristic**	**K10**			**Days unable to work**		
	**Mean**	**Std.** ** Dev**.	***P* value**	**Mean**	**Std.** ** Dev**.	***P* value**
**Gender**						
Male	22	9.1	<0.001	5.9	7.7	<0.001
Female	24.6	9		7.3	7.6	
**Change place of living due to war**						
No	23.4	9.1	<0.001	6.8	7.7	0.381
Within the same city	24.4	9		7.2	7.6	
To another city	24.8	9.2		7.1	7.6	
**Distress from war noises**						
No	22.5	9.2	<0.001	6.2	7.8	0.005
Yes	24.1	9.1		7.1	7.6	
**Being a student**						
No	23	9.1	<0.001	5.1	6.8	<0.001
In a school	25.3	9.5		8.4	7.3	
In a university of high institute	24.5	9		8.2	8.1	
**Education**						
Elementary	24.5	10	0.001	4.9	7.2	<0.001
Until grade 9	23.4	9.4		4.5	5.9	
High School	24.4	9.3		8	8.3	
University, or high institute	24	9.1		7	7.7	
Master or higher	22.4	8.7		5.3	6.4	
**House that you currently live in**						
Owned	23.6	9.1	0.002	6.8	7.5	0.194
Rented or given by the government	24.1	9		7.1	8	
Living in friends\relatives house	25	9.5		7.3	8	
**Chronic Medical Condition**						
No	23.3	9.3	<0.001	6.5	7.5	0.005
Hypertension	24.6	10.1		6.5	8.2	
Diabetes	22.1	9.6		5.1	6.6	
Asthma	26.3	9.2		8.3	8.4	
Other	26.6	9.3		8	8.1	
Two or more	25.1	10.1		8	8.7	
**Chronic Condition With Housemates**						
No	23	9.5	<0.001	6.3	7.4	0.004
Hypertension	23.7	8.8		7	7.7	
Diabetes	23.7	8.8		8.2	8.5	
Asthma	25.2	9.2		7.1	7.6	
Other	25.8	8.8		7.7	8.2	
Two or more	25.3	9.3		7.5	7.9	
**Marital Status**						
Single	24.2	9	<0.001	7.5	7.8	<0.001
In a relationship	25.2	9.5		8.5	8.5	
Married	22.6	9		4.9	6.5	
Divorced	21.6	8.9		5.7	8	
Engaged	25	9.6		7.5	8	
Widowed	22.8	8.6		4.9	6.8	
**Smoking cigarette or shisha**						
I was not a smoker and did not start	23.7	8.9	<0.001	6.9	7.5	0.431
I used to smoke one of them but now I smoke both	26.8	9.2		8.6	9	
Yes I am smoking now and before COVID 19	24.2	9.5		7	8	
I started smoking cigarettes	25.5	12.3		8.4	8.3	
I started Shisha	27.8	10.9		7	8.2	
**SmokingChangesFromCOVID19**						
Stopped smoking	22.7	9.1	<0.001	6.8	8.7	0.006
Almost the same	22.7	9.2		6.1	7.8	
Increased dramatically	27.4	10.1		8.5	8.7	
Decreased dramatically	23.9	9		6.9	8.2	
**Monthly Income**						
It cannot cover buying the essentials	25.4	9.8	<0.001	7.9	8.4	<0.001
It is only enough for essentials from food and drink	23.6	8.8		6.8	7.4	
It is enough for essentials and other things	22.6	8.8		6.2	7.2	
**Have you been suspected of having COVID-19**						
No symptoms or signs	23.8	9.1	0.002	6.9	7.7	0.686
Yes, has some possible symptoms, but no diagnosis	26.9	9.1		7.6	7.3	
Yes, I was diagnosed with COVID-19 by a doctor	17	.		7	.	
**Exposed to someone likely to have COVID-19**						
No	23.7	9.1	<0.001	6.9	7.6	0.007
Yes, has some possible symptoms, but no diagnosis by doctor	26.7	8.9		7.6	7.8	
Yes, with someone was diagnosed by a doctor	26.3	9.7		15.4	10.3	
**Anyone in your family been diagnosed with COVID-19**						
No	23.8	9.1	0.96	6.9	7.7	0.329
Yes, non-household member	23.3	8.4		7.7	7.5	
Yes, member of household	23.9	8.1		9.4	9.3	
**Happened because of COVID-19:**						
**Fallen ill physically**						
No	23.5	9.1	<0.001	6.7	7.5	<0.001
To a family member	26.3	8.9		8.9	8.7	
To a housemate	26.5	8.8		6.5	7.1	
To you	26.4	8.5		8.3	7.7	
**Hospitalized**						
No	23.8	9.1	0.009	6.9	7.7	0.821
To a family member	26.3	9.3		7.8	7.7	
To a housemate	25.8	10		7.5	6	
To you	29.3	9.6		7.4	8.6	
**Self-quarantine with symptoms**						
No	23.8	9.1	0.28	6.9	7.7	0.382
To a family member	27.4	8.8		7.6	6.1	
To a housemate	27.1	5.6		11.9	7.2	
To you	27.6	6.8		7.6	8.2	
**Self-quarantine without symptoms**						
No	23.8	9.1	0.054	6.9	7.7	0.496
To a family member	27.8	8.9		9.3	9.8	
To a housemate	30.1	5.4		9.8	7.4	
To you	25.3	8.6		6.9	7.1	
**Lost Job**						
No	23.2	8.9	<0.001	6.6	7.5	<0.001
To a family member	25.5	9		8.1	8	
To a housemate	25.4	9.9		6	7	
To you	24.1	9.3		6.6	7.5	
Two of the previous	25.4	9.4		7.3	8.1	
All of the previous	26.9	10.3		8.6	8.5	
**Reduced ability to earn money**						
No	22.9	8.9	<0.001	6.3	7.3	<0.001
To a family member	25.5	9		8.3	8.1	
To a housemate	26.1	9.2		6.2	6.8	
To you	24.1	9.4		6.5	7.6	
Two of the previous	25.8	9.2		7.9	8.4	
All of the previous	27.8	10.1		9.2	8.9	
**Passed away**						
No	23.8	9.1	0.003	6.9	7.7	0.242
To a family member	29.5	9.9		9.6	7.7	
To a housemate	29.3	6.2		10	4.1	
**How worried have you been from:**						
**Being infected**	22.1	9.6	<0.001	6.9	8.3	<0.001
Not at all	22.8	8.5		6.4	7.3	
Slightly	24.9	8.6		7.1	7.5	
Moderately	28.9	9.2		8.5	8.3	
Very						
**Friends or family being infected**						
Not at all	19.9	9.4	<0.001	5.1	7.6	<0.001
Slightly	21.8	8.5		6.1	7.4	
Moderately	23.3	8.4		6.8	7.3	
Very	26.5	9.3		8	8	
**Your work will be negatively affected**						
Not at all	22.5	8.7	<0.001	6.8	7.8	0.006
Slightly	23.1	8.6		6.3	7.1	
Moderately	23.4	8.9		6.8	7.4	
Very	26.7	9.5		7.5	7.9	
**Your studies will be negatively affected**						
Not at all	22.8	8.9	<0.001	5.8	7.5	<0.001
Slightly	22.9	8.4		6.7	7.4	
Moderately	23.5	8.4		7.7	7.3	
Very	27.1	9.6		9.4	8.4	
**Providing food will be affected**						
Not at all	21.4	8.6	<0.001	6	7.4	<0.001
Slightly	22.5	8.3		6.4	7.2	
Moderately	24.2	8.6		7.3	7.9	
Very	27.4	9.7		8.2	8.1	
**Are you committed to quarantine**						
I do not go out at all	24.4	9.1	0.009	7.9	7.9	<0.001
Only for essentials	23.5	9.1		6.6	7.5	
Reduced my going out	24	9.2		6.2	7.4	
Have not changed at all	25.1	10.2		5.4	7.7	
**Quality of your relationships with your friends**						
A lot better	24.3	9.1	<0.001	6.5	6.2	<0.001
A little better	24.3	9.2		7.2	7.1	
About the same	23	8.9		6.4	7.5	
A little worse	26.3	9		8.2	7.9	
A lot worse	27	10.1		9.6	8.8	
**Quality of your relationships with your housemates**						
A lot better	21.7	8.7	<0.001	5.3	6.4	<0.001
A little better	23.4	8.6		6.4	7	
About the same	22.4	8.6		6.3	7.5	
A little worse	28.7	8.5		9.3	8	
A lot worse	34.2	9.1		12.9	9.1	
**Quality of the relationships between you and members of your family**						
A lot better	21.9	8.3	<0.001	5.8	7.2	<0.001
A little better	23.4	8.8		6.7	7.5	
About the same	23.1	8.8		6.5	7.5	
A little worse	27.3	9.1		8.5	7.9	
A lot worse	29.2	10.9		11	9.2	
**Quality of your relationships with your Partner**						
A lot better	22.1	9.6	<0.001	5.1	6.7	<0.001
A little better	23.6	8.7		5.9	6.8	
About the same	22	8.7		5.4	6.8	
A little worse	28	8.7		9	8.5	
A lot worse	32	9.5		10.8	8.5	
**Drugs increases after COVID-19**						
No drugs	22.6	8.7	<0.001	6.5	7.5	0.002
Analgesics and NSAIDS	26.8	9.1		7.7	7.8	
OTC flu medications	23.8	8.3		6.5	7.3	
Hypnotics	32.8	9.8		9.5	8.9	
Antibiotics	24.7	9.3		7.6	7.5	
Other	26.1	9.6		6.6	8	
2 drugs	27.9	9.9		6.8	7.5	
3 drugs or more	27.7	10.2		9	8.1	
**Drugs increases for your family**						
No drugs	22.9	9	<0.001	6.5	7.6	<0.001
Analgesics and NSAIDS	25.9	8.9		7.6	7.6	
OTC flu medications	25.1	8.4		6.9	7.3	
Hypnotics	27.6	9		12.2	7.7	
Antibiotics	27.7	8.7		9.3	8.1	
Other	26	9.5		7.4	8.7	
2 drugs	24.1	8.6		8.6	8.9	
3 drugs or more	27.3	8.1		9.2	9.6	

**Table 6:** Correlations between each cluster of MSPSS, SPTSS, and K10.

	**Significant other support**	**Family Support**	**Friends support**	**Total MSPSS Score**
Avoidance	*r* = −0.213	*r* = −0.242	*r* = −0.195	*r* = −0.269
Arousal	*r* = −0.137	*r* = −0.190	*r* = −0.157	*r* = −0.199
Re-experience	r = −0.171	*r* = −0.215	*r* = −0.175	*r* = −0.231
Total SPTSS score	*r* = −0.207	*r* = −0.256	*r* = −0.209	*r* = −0.278
K10 total score	*r* = −0.164	*r* = −0.230	*r* = −0.166	*r* = −0.229
Days unable to work	*r* = −0.169	*r* = −0.212	*r* = −0.147	*r* = −0.220
Age	*r* = 0.124	*r* = 0.080	*r* = 0.033^*^	*r* = 0.100
	**Avoidance**	**Arousal**	**Re-experience**	**Total SPTSS score**
K10 total score	*r* = 0.600	*r* = 0.594	*r* = 0.553	*r* = 0.683
Days unable to work	*r* = 0.400	*r* = 0.327	*r* = 0.330	*r* = 415
Age	*r* = −0.113	*r* = −0.079	*r* = −0.073	*r* = −0.104

*All correlations were significant at P < 0.001*.

* *P = 0.016*.

*Correlation between K10 total score and days of unable to work was not significant (r = 0.021 and P = 0.159)*.

### PTSD

Avoidance, arousal, and total PTSD scores differed according to governorate (*P* = 0.009, *P* = 0.060, and *P* = 0.020, respectively). These PTSD scores differences are demonstrated in Figure 1. All PTSD items did not correlate with consanguinity (*P* > 0.05). PTSD items and total scores differed according to governorate and type of work or being unemployed (*P* < 0.05), with total PTSD score differences having (*P* < 0.001) and demonstrated in Figure 1.

Regressing gender, educational level, rented housing, type of work, having a chronic medical condition, marital status, monthly income adequacy, distress from war noises, and changing place of living due to war on PTSD scores using forward linear regression was significant (*P* < 0.001) with having a chronic condition (R^2^ = 3.2%), gender (R^2^ = 1.4%), monthly income adequacy (R^2^ = 1.3%), marital status (R^2^ = 0.3%), changing place of living due to war and educational level (R^2^ = 0.2%) contributing to the variance. SPTSS item score correlations are demonstrated in Table 3.

### MSPSS

Family, friends, significant other, and total support were significantly correlated with the type of work and governorate (*P* < 0.001), but with consanguinity (*P* > 0.05).

Regressing gender, educational level, rented accommodation, type of work, having a chronic medical condition, marital status, monthly income adequacy, distress from war noises, and changing place of living due to war on total MSPSS score using forward linear regression was significant (*P* < 0.001), with social status (R^2^ = 2.4%), monthly income adequacy (R^2^ = 1.8%), having a chronic medical condition (R^2^ = 1.3%), and educational level (R^2^ = 1%) while living in rented accommodation, type of work, and distress from war noises (R^2^ = 0.2%) and (*P* < 0.05) contributing to the variance.

### K10

K10 had no association with consanguinity (*P* > 0.05). K10 total score was different according to governorate and type of work or being unemployed (*P* < 0.05), with total PTSD score differences having (*P* < 0.001) and demonstrated in Figure 1.

When Regressing gender, educational level, rented accommodation, type of work, having a chronic medical condition, marital status, monthly income adequacy, distress from war noises, and changing place of living due to war on total K10 score by using forward linear regression, (*P* < 0.001) for gender (R^2^ = 2.2%), and having a chronic condition (1.7%), monthly income adequacy (R^2^ = 0.8%), and social (R^2^ = 0.6%) while change place of living due to war (R^2^ = 0.4%) and educational level (R^2^ = 0.3%) for (*P* < 0.05). When using forward linear regression on total days of not being able to work with the same previous variables, (*P* < 0.001) for the type of work (R^2^ = 1.5%), gender (R^2^ = 0.8%), marital status (R^2^ = 1%), having a chronic medical condition (R^2^ = 0.8%), and monthly income adequacy (R^2^ = 0.6%).

### COVID-19 Variables

When regressing gender, educational level, rented accommodation, type of work, having a chronic medical condition, marital status, monthly income adequacy, distress from war noises, changing place of living due to war, along with other COVID-19 variables of distress from losing the job, decreased income, passing away, the distress of being infected or a family member, distress of the job, studies, and food being affected on total SPTSS score by using forward linear regression, (*P* < 0.001) for the distress that the ability to provide food will be affected (R^2^ = 8%), distress from friend or family being infected (R^2^ = 2.6%), having a chronic condition (R^2^ = 1.9%), studies being affected (R^2^ = 1.6%), gender (R^2^ = 1.4%), job being affected (R^2^ = 1%), and passing away (R^2^ = 0.6%) while losing their job, being infected (R^2^ = 0.4%), and educational level [R^2^ = 0.2%, *P* < 0.05].

When regressing the same previous variables on total MSPSS score, (*P* < 0.001) for social status (R^2^ = 3.3%), monthly income adequacy (R^2^ = 1.6%), having a chronic condition (R^2^ = 1%), passing away (R^2^ = 0.8%), and educational level (R^2^ = 0.7%) while decreased income, type of work, and distress from work noise [R^2^ = 0.3%, *P* < 0.05]. When regressing the same previous variables on total K10 score with the same previous variables, (*P* < 0.001) for the distress that the ability to provide food is affected (R^2^ = 6.6%), gender (R^2^ = 2.5%), studies being affected (R^2^ = 1.7%), a friend or family being infected (R^2^ = 1.3%), having a chronic medical condition (R^2^ = 1%), and losing their job (R^2^ = 0.7%) while job being affected (R^2^ = 0.3%), educational level, distress being infected and passing away [R^2^ = 0.2, *P* < 0.05). When regressing the same previous variables on total days of not being able to work with the same previous variables, (*P* < 0.001) for the distress that studies will be affected (R^2^ = 3%), gender (R^2^ = 1%), the distress of food provision being affected (R^2^ = 0.9%), and type of work (R^2^ = 0.8%), while having a chronic medical condition (R^2^ = 0.6%), marital status (R^2^ = 0.3%), and decreased income (R^2^ = 0.4%), and educational level (R^2^ = 0.3%) for (*P* < 0.05).

No significant difference between gender and smoking changes in lockdown (*P* > 0.05). However, female participants felt more distress from war noises, more symptoms in the last two weeks, worried more than a friend or member of their family might have it, worried less that job will be affected or the ability to provide food will be affected, committed more to lockdown, their relationships worsened less with friends but more with family in the house and other family members, and took more medication than male participants (*P* < 0.05). No significant difference between gender in the relationship with partner or studies being affected (*P* > 0.05).

Being single was correlated with decreasing amount smoked in lockdown more (*P* < 0.011) and was correlated with less distress from war (*P* < 0.001). Being married was correlated with less distress of family or friend having COVID-19 when compared to being single (*P* < 0.001), but with more distress of job being affected (*P* = 0.009). There was no significant difference in distress about not being able to provide food during the COVID-19 pandemic and being single or married (*P* > 0.05).

Younger ages were insignificantly correlated with being distressed from acquiring COVID-19 (*P* = 0.082), significantly correlated with being distressed that a family may have it, a job affected, studies, and providing food being affected from COVID-19 (*P* < 0.05). Relationships with friends was more stable in older ages as in the younger ages they tended to improve or deteriorate (*P* < 0.001) while the relationship with housemates was not affected dramatically Younger participants overall their relationship deteriorate more frequently (*P* < 0.001). Moreover, the relationship with other family members and partners improved more frequently in the older ages (*P* < 0.001).

## Discussion

Our study found that 1,891 (35.6%) participants had avoidance symptoms, 2,558 (48.7%) had arousal symptoms, 2,305 (44.3%) had re-experience symptoms, 1,005 (19.4%) had two PTSD symptoms, 2,301 (42.6%) had moderate to severe mental disorder, and 706 (14.9%) had low social support. Furthermore, female participants were found to have higher avoidance, arousal, re-experience, and mental disorder (K10) scores despite having higher social support scores. Moreover, being distressed from war noises, changing their place of living due to war, being a university or school student, not having an adequate monthly income, and most COVID-19 worries were associated with increased SPTSS and K10 scores and a decreased social support score. However, when regressing to determine the most significant contributing factors, we found that distress about providing food was the main contributing factor to the high SPTSS and K10 scores followed by distress from friends or family being infected, studies being affected from COVID-19, their job being affected from COVID-19, and gender. Social status and monthly income adequacy were the highest contributing factors to having low social support. Interestingly, no factors related to war were found to be significant when using the regression model.

### COVID-19

This study assessed the psychological distress caused by the lockdown, and the new social and financial challenges caused by the pandemic, rather than by the illness itself, as <10 cases were confirmed in Syria at the time of the study. Our results show that psychological distress and PTSD symptoms are more common in participants with weak social support, problems at work, and who face challenges in the provision of food. Although our study showed a decrease in the overall frequency of smoking in both genders, there was no significant change in smoking habits among genders This can be explained by an increase in the cost of tobacco during the lockdown or could because of warnings of the effects of smoking on COVID-19.

Regular habits and war exposure in Syria were found to be related to unusual exposures to different substances (19). This exposure has led to an increase in different medical conditions compared to other countries such as allergic rhinitis (20) and laryngopharyngeal reflux disease (21), and both were related to distress from war noises.

Regarding familial relationships, almost half of the participants reported a difference in the relationship with their housemates but over 70% did not report a change in the relationship with other family members and friends.

### PTSD, MSPSS, and K10 With COVID-19

#### Our Study

The provision of food during the lockdown was a big stressor identified by the participants, associated with higher SPTSS and K10 scores. Higher PTSD and mental disorder scores were observed in subjects with a deteriorated relationship with their family, housemates, and friends. This could be from the impact of reduced supports for mental health or from participants with higher PTSD and mental disorder scores suffering from more fragile relationships.

Although participants who tested positive for COVID-19 were few, higher PTSD and mental disorder scores were seen amongst them and their family members. A quarter of the participants did not report having flu-like symptoms but those who had symptoms had not been diagnosed. Higher scores of PTSD and mental distress were also observed in subjects who were self-isolated when asymptomatic compared to an asymptomatic family member who had not. Higher scores were seen when a housemate's ability to earn money was affected, more than when a family member's ability to earn money was affected. This could be explained by the fact that female participants comprised over two-thirds of our sample, and in Syria, a large number of women rely on their housemates to earn money.

Lower PTSD and mental disorder scores were observed in those that ignored lockdown instructions and were able to go out and buy household essentials. Patients with higher scores reported using hypnotics more frequently. Interestingly, social support decreased in participants who did not smoke or used to smoke and commenced using shisha which could be from this habit affecting housemates or the irritability from ceasing cigarette smoking. Higher social support was found in participants with relationships not being affected or improved in the lockdown while higher PTSD scores were seen in participants whose relationships deteriorated with their partners.

Fewer working hours being affected from mental distress were observed in married participants which could be explained by schools being closed and parents having to stay home to look after their children. Higher mental disorder scores and fewer working days being affected from mental distress were observed in participants who increased their smoking habits. Furthermore, the higher the K10 score, the more symptomatic the participants became. Interestingly, subjects with decreased working hours had higher social support and therefore a better relationship status. Higher PTSD scores were observed with higher mental disorder scores, and more days of being unable to work with correlations of (*r* = 0.682), and (*r* = 0.415), respectively. Interestingly, mental disorders scores and days of being unable to work had an insignificant correlation (*P* = 0.159).

#### Other Studies

The nature of COVID-19 required new methods to collect data as it can be expensive to safely collect data from a large population. The impact that the pandemic has is still uncertain, which could cause anxiety, worry, and even despair (22). Internet-based questionnaires proved to be very effective during the pandemic in many countries where specific questionnaires were developed for this purpose, for example in Italy (23).

Many studies have also found that COVID-19 had severe effects on mental health. Studies from Italy indicated that COVID-19 was linked to anxiety, distress, and symptoms of PTSD (24, 25). The lockdown also affected sleep quality (24). An association was found with general anxiety, and depression in Ireland (26). Another study from Hong Kong found that around a quarter of the population declared that their mental health got worse during the pandemic. Most of the worries were from being infected, not having sufficient masks, and being bothered when not being able to work from home. Interestingly, an association with a previous event was established as participants who did not have experience with the previous SARS outbreak had a worse mental health status (27).

One study among adults in the USA found that COVID-19-related experience was associated with an odds ratio higher than 3 of having probable anxiety and depression and the stress from COVID-19 could predict a variance of (R^2^ ≥ 30) with anxiety and depression (28). However, a study in Spain demonstrated that the levels of symptoms of depression, anxiety, and stress were low at the beginning, but these levels rose after staying at home (29). Nevertheless, a study in the UK found that the early stages of the pandemic were associated with a modest increase of mental health problems including anxiety, depression, and trauma symptoms (30).

### PTSD, MSPSS, and K10 With Other Variables

As PTSD needs at least 4 weeks to develop, we used the SPTSS score as a probable indicator rather than cut-off points. Having certain medical conditions was associated with higher scores in SPTSS and K10, especially when having a family member with chronic medical conditions like asthma. This may indicate the need to address the psychological effect of chronic medical conditions, not only on the affected person but on other members of their household. Participants who did not own the house they lived in and had an inadequate monthly income had higher SPTSS and K10 scores which indicates the role of financial burden. Avoidance scores were higher when losing a housemate while other PTSD symptoms scores increased more when participants had lost a family member. This could be explained by the stereotype brought to the widowed woman in the community that makes her feel isolated. Another theory could be that the morale of the widowed dropped, as losing a partner is considered a major trauma and the possibility of losing the social and financial support of the partner is also significant.

Mental distress and PTSD have been identified in many studies to be more common in female participants, particularly from war noises (12). Distress was also more common in female schoolchildren aged 15 years and over, but male schoolchildren had more tendency to smoke (31), which was similar to adults in Syria (32). The high prevalence of PTSD and severe mental distress are not novel findings and the prevalence found in our study was lower than a previous study that used the same materials and same scales conducted a year ago (12). The previous study found that 60.8% of the sample had two positive PTSD clusters or more according to DSM IV and 61.2% of the sample had moderate to severe mental disorders according to K10. Our study findings were also lower than those of a study on Syrian schools in Damascus (31) in which PTSD prevalence was found to be 53%. This may indicate that war may have a more severe effect than the pandemic.

Many risk factors for psychological distress were identified that were associated with COVID-19 such as female gender, being younger than 50 years of age, being in direct contact with someone who was infected by COVID-19, having a high risk of acquiring COVID-19, the presence of children at home, low income, having previous medical conditions in them or with others and being uncertain about the risk of contagion (25, 26, 30). However, despite the finding that psychological distress is associated with younger age, being 65 years or older was associated with more severe anxiety related to COVID-19 (26). In Turkey, a neighboring country to Syria, it was found that being female, living in an urban place, and having a previous psychiatric problem were associated with anxiety, compared to depression which was only associated with living in an urban place. However, being female, having a chronic medical condition, and having psychiatric disorders were associated with anxiety (33).

## Limitations

As PTSD needs four weeks to develop (according to the strict definition), we used scoring symptoms rather than diagnosing cut-off, which is perhaps more indicative of an acute stress disorder. This study was online and mainly involved participants who had enough free time to fill in the survey which may neglect the truly affected population. Furthermore, the days of fewer productivity questions in K10-LM may not be fully understood for some participants and therefore were not mentioned in the discussion. This study used a questionnaire and is not based on medical diagnosis, which would have been more accurate. No clinical examination by professionals was conducted and therefore determining if the effect of COVID-19 was truly more severe than war is not conclusive.

The sampling method, the use of an online questionnaire, the sample size compared to the population, and the subjectivity of the used method were the main source of biases in our study.

## Conclusion

Although full lockdown can be an effective method for preventing the spread of pandemic diseases such as COVID-19, this study indicates that it causes severe distress. This is reflected in mental distress and PTSD symptoms even in countries that were originally affected by wars, as the restrictions of lockdowns can obstruct lifestyle and the ability to earn and provide daily food. Social support has a role and to some extent reduces the amount of stress either on PTSD or distress from COVID-19. Being widowed, low SES, low social support, and living in rented accommodation were associated the most with distress as the ability to earn and to provide food were the most common stressors. Having high K10 and PTSD scores were associated with having more symptoms of COVID-19 despite not being exposed to it. PTSD and severe mental distress were prevalent in the Syrian community due to the psychological effects of war in the previous 9 years. Positive PTSD clusters in this study were less prevalent compared to previous studies in Syria which may suggest that the first weeks of lockdown might reduce the stress. However, this study suggests that COVID-19 stress, mainly from the effect on the economy, might be more distressing than experiencing war.

## Data Availability Statement

The raw data supporting the conclusions of this article will be made available by the authors, upon reasonable request.

## Ethics Statement

Informed consent was taken before participants completed the survey. Informed consent was also taken for using and publishing the data. Confidentiality was assured by not asking or publishing any data that may refer to the individual identity. The ethical aspects of the study were approved by Damascus University deanship (Damascus, Syria).

## Author Contributions

AK: conceptualization, data curation, formal analysis, investigation, methodology, project administration, supervision, resources, validation, original draft, writing, review, and editing. AF: review and editing, original draft, investigation, software, and resources. LM: original draft, writing, review, and editing. AG: conceptualization, project administration, editing, and software. RA: project administration, investigation, and resources. All authors have read and approved the manuscript.

## Conflict of Interest

The authors declare that the research was conducted in the absence of any commercial or financial relationships that could be construed as a potential conflict of interest.

## Abbreviations

ANOVA: Analysis of variance
CI: Confidence Interval
COVID-19: Coronavirus disease 2019
CRISIS: The CoRonavIruS Health Impact Survey
DSM: Diagnostic and statistical manual of mental disorders
ICD: International Classification of Disease
K10: Kessler
MSPSS: Multidimensional Scale of Perceived Social Support
NMIH: National Institute of Mental Health
PTSD: post-traumatic stress disorder
SES: Socioeconomic status
SPSS: Statistical Package for the Social Sciences
SPTSS: Screen for Posttraumatic Stress Symptoms.
